# A phylogenomic analysis of Marek's disease virus reveals independent paths to virulence in Eurasia and North America

**DOI:** 10.1111/eva.12515

**Published:** 2017-09-03

**Authors:** Jakob Trimpert, Nicole Groenke, Maria Jenckel, Shulin He, Dusan Kunec, Moriah L. Szpara, Stephen J. Spatz, Nikolaus Osterrieder, Dino P. McMahon

**Affiliations:** ^1^ Institut für Virologie Freie Universität Berlin Berlin Germany; ^2^ Institute of Diagnostic Virology Friedrich‐Loeffler‐Institut Greifswald‐Insel Riems Germany; ^3^ Institut für Biologie Freie Universität Berlin Berlin Germany; ^4^ Department for Materials and Environment BAM Federal Institute for Materials Research and Testing Berlin Germany; ^5^ Department of Biochemistry and Molecular Biology Center for Infectious Disease Dynamics and the Huck Institutes of the Life Sciences Pennsylvania State University University Park PA USA; ^6^ United States Department of Agriculture US National Poultry Research Center Athens GA USA

**Keywords:** disease, emergence, evolution, resistance, virulence

## Abstract

Virulence determines the impact a pathogen has on the fitness of its host, yet current understanding of the evolutionary origins and causes of virulence of many pathogens is surprisingly incomplete. Here, we explore the evolution of Marek's disease virus (MDV), a herpesvirus commonly afflicting chickens and rarely other avian species. The history of MDV in the 20th century represents an important case study in the evolution of virulence. The severity of MDV infection in chickens has been rising steadily since the adoption of intensive farming techniques and vaccination programs in the 1950s and 1970s, respectively. It has remained uncertain, however, which of these factors is causally more responsible for the observed increase in virulence of circulating viruses. We conducted a phylogenomic study to understand the evolution of MDV in the context of dramatic changes to poultry farming and disease control. Our analysis reveals evidence of geographical structuring of MDV strains, with reconstructions supporting the emergence of virulent viruses independently in North America and Eurasia. Of note, the emergence of virulent viruses appears to coincide approximately with the introduction of comprehensive vaccination on both continents. The time‐dated phylogeny also indicated that MDV has a mean evolutionary rate of ~1.6 × 10^−5^ substitutions per site per year. An examination of gene‐linked mutations did not identify a strong association between mutational variation and virulence phenotypes, indicating that MDV may evolve readily and rapidly under strong selective pressures and that multiple genotypic pathways may underlie virulence adaptation in MDV.

## 1 INTRODUCTION

Understanding the evolution of virulence is a major focus of pathogen research and has important ramifications for evolutionary theory, host–parasite ecology, and epidemiology (Alizon, Hurford, Mideo, & Van Baalen, 2009; Cressler, McLeod, Rozins, van den Hoogen, & Day, 2016; Ebert, 1998; Schmid‐Hempel, 2008, 2009, 2011; Woolhouse, Taylor, & Haydon, 2001). Virulence is a key trait of any pathogen, and it is governed by many factors including transmissibility. Understanding virulence evolution is critical for addressing some of the global challenges posed by pathogens to humans, livestock, companion animals, and wildlife, be it the (re‐) emergence of infectious diseases (Daszak, 2000; Hawley et al., 2013; Morens, Folkers, & Fauci, 2004; Morse et al., 2012; Woolhouse, Haydon, & Antia, 2005) or the evolution of therapeutic resistance (Carroll et al., 2014; Gandon, Mackinnon, Nee, & Read, 2001; Read et al., 2015; Woolhouse & Ward, 2013). Despite the importance of virulence evolution, the prevailing theory, which is based on a life‐history trade‐off framework (Anderson & May, 1982; Ewald, 1983), has to some extent suffered from a lack of empirical validation (Cressler et al., 2016). In particular, the causal link between virulence and transmission has come under scrutiny (Bull & Lauring, 2014; Ebert & Bull, 2003).

The debate has also been reinvigorated following recent research into the evolution of Marek's disease virus (MDV, also referred to as *Gallid herpesvirus 2* [GaHV‐2]), which is a member of the genus *Mardivirus* in Herpesviridae (subfamily Alphaherpesvirinae). MDV infects chickens and rarely other avian species and is controlled in the global poultry industry by the near‐universal application of modified‐live virus vaccines, without which infected chickens typically develop acute rash, and edematous neuronal and brain damage: severe lymphomas, paralysis, and death at a very young age (Witter, 1997, 2001). The severity of disease in unvaccinated chickens has steadily increased since the adoption of large‐scale farming techniques in the 1950s and mass vaccination since the 1970s (Nair, 2005; Osterrieder, Kamil, Schumacher, Tischer, & Trapp, 2006; Witter, 1998). Data from a recent study show that newer MDV lineages that evolved in the vaccination era are significantly fitter than ancestral vaccine‐naïve strains (Read et al., 2015). These findings support theoretical predictions (Gandon et al., 2001; Smith & Mideo, 2017) that the use of vaccines to suppress but not block pathogen replication or transmission (so called antidisease, imperfect, or leaky vaccines) results in the evolution of viruses with increased replication (i.e., fitness) or transmission and therefore virulence. Meanwhile, previous studies addressing the industrialization of farming have discussed how MDV virulence can increase independently of vaccine use (Atkins, Read, Walkden‐Brown et al., 2013; Atkins, Read, Savill et al., 2013; Rozins & Day, 2017). In these studies, denser flocks and longer durations for rearing in combination with shorter intercohort intervals and limited virus elimination by cleaning and disinfection can lead to reduced MDV virulence. Many of these factors seem counterintuitive from the perspective of disease prevention, but they indicate how substantially shortened host cohort durations, which have halved for broiler chickens in 60 years due to advances in nutrition and breeding (Anthony, 1998), could have inadvertently contributed to the evolution of increased virulence.

Both vaccine use and intensive farming can influence pathogen transmission dramatically because they artificially manipulate the immune status and population dynamics of the host. The impact of these factors on transmission could in principle result in a drive toward increased pathogen virulence. Given that both imperfect vaccination and industrialized farming practices are now pervasive in the global poultry industry, we set out to explore their impact on the evolution of MDV. We present findings from a phylogenomic study aimed at resolving the geographical, temporal, and ancestral trait patterns of MDV virulence evolution in the context of these changes.

## 2 METHODS

### 2.1 Genome sequencing

Viral DNA was obtained from the blood of infected animals or from chicken embryo cells (CEC) that were infected with the respective strain of MDV as described earlier (Schumacher, Tischer, Fuchs, & Osterrieder, 2000). None of the viruses were passaged more than five times on CEC. For DNA extraction, 200 μl buffy coat from infected animals or infected CEC (at day 7 postinfection) from a 100‐mm dish was resuspended in 500 μl TEN buffer (100 mm NaCl, 10 mm Tris, 1 mm EDTA, pH 8.0) and lysed by the addition of 250 μl sarcosine lysis buffer (75 mm Tris, 25 mm EDTA, 3% (w/v) N‐lauryl sarcosine, pH 8.0) followed by a 15‐min incubation at 65°C. RNA was degraded by the addition 10 μl RNAse A (10 mg/ml) and 30‐min incubation at 37°C. Protein was digested during a 16‐hr incubation after the addition of 10 μl proteinase K (20 mg/ml). DNA was extracted using a standard phenol/chloroform procedure followed by ethanol precipitation (Sambrook, Fritsch, & Maniatis, 1989). After centrifugation, DNA was resuspended in 50 μl TE buffer and DNA concentration determined by spectrophotometry.

Five micrograms of total DNA was diluted in 130 μl TE and fragmented to a peak fragment size of 500–700 bp using the Covaris M220 focused ultrasonicator (peak incident power: 75 W, duty cycle: 5%, cycles per burst: 200, duration: 52 s). Fragmented DNA (1 μg) was used for NGS library preparation using the NEBNext Ultra II Library Prep Kit for Illumina platforms (New England Biolabs).

To capture viral sequences from total cellular DNA extracts, we developed a tiling array using a bait set designed to capture the sequence of MDV strain RB‐1B (GenBank EF523390.1). The tiling array consisted of approximately 6,200 biotinylated RNA 80‐mers, employed according to manufacturer's instructions (Mycroarray, Ann Arbor, MI). Highly enriched viral sequence libraries were generated from 500 ng of indexed total DNA libraries. For sequencing on a MiSeq instrument (Illumina), target‐enriched libraries were quantified by qPCR using the NEBNext Library Quant Kit (New England Biolabs). Up to 48 samples were pooled to equal molarity and subjected to sequencing using Illumina's v3 chemistry for 2 × 300 bp paired‐end sequencing. Read quality of NGS data was assessed with FastQC (Andrews, 2010). Reads were preprocessed by Trimmomatic (Bolger, Lohse, & Usadel, 2014) to remove adapters, low‐quality regions, and reads <36 bp. Alignment was performed using BWA (Li & Durbin, 2009) against the reference genome of the very virulent (vv) MDV strain Md5 (RefSeq NC_002229.3), which consists of 177,874 bp and encodes 103 predicted proteins (Tulman et al., 2000). Freebayes (Garrison & Marth, 2012) and BCFtools (Li et al., 2009) were then used to create a consensus sequence of mapped reads.

### 2.2 Evolutionary analysis

We combined four newly sequenced consensus genomes with 18 complete or near‐complete MDV genomes obtained from GenBank (for detailed strain information, see TableS1). The genomic sequences were aligned using MAFFT v7.205 (Katoh & Standley, 2013). For phylogenetic analysis, alignment gaps associated with incomplete genomic data, variable repeat regions, mini‐F vector sequences, and reticuloendotheliosis virus long terminal repeats (the latter two being present in some of the full‐genome sequences) were removed using Gblocks v0.91 (Castresana, 2000). Remaining ambiguous nucleotide positions were treated as missing data in subsequent analyses. Genomic variation was visualized with Nei's Pi using a 1‐kb sliding window and 100‐bp interval length in the package PopGenome v2.6.1 (Pfeifer, Wittelsburger, Ramos‐Onsins, & Lercher, 2014) within the R programming suite v3.1.3 (R Core Team, 2016). Synonymous, nonsynonymous, and insertions/deletions (indels) in predicted open‐reading frames (ORFs) derived from the Md5 reference genome were quantified using MEGA v5.2.1 (Tamura et al., 2011). Maximum‐likelihood (ML) phylogenetic trees were constructed using PhyML v3.0 (Guindon et al., 2010) with 1000 bootstrap replicates, using a generalized time‐reversible (GTR) site substitution model plus Gamma (Γ_4_) distribution and nearest neighbor interchange for tree topology improvement. Temporal signal was examined by plotting root‐to‐tip phylogenetic divergences using TempEst v1.5 (Rambaut, Lam, Max Carvalho, & Pybus, 2016). We employed RDP, GENECONV, and BootScan within the software package RDP4 v4.56 (Martin, Murrell, Golden, Khoosal, & Muhire, 2015) to detect evidence of recombination, using a threshold of *p* < .05 and Bonferroni correction to account for multiple testing. Recombinant events were accepted if they were detected by all three methods. The impact of recombination on the MDV phylogeny was also explored by comparing topologies and temporal signals of trees reconstructed either with or without the identified recombinant regions.

A final alignment of 20 genomic sequences was analyzed in a clock‐based phylogenetic framework in BEAST v1.8.4 (Drummond, Suchard, Xie, & Rambaut, 2012) under a range of substitution (GTR + Γ_4_, Hasegawa, Kishino, and Yano (HKY) (Hasegawa, Kishino, & Yano, 1985)  + Γ_4_), demographic (constant, exponential), and molecular clock evolutionary models (strict, relaxed uncorrelated lognormal). Where applicable, a continuous‐time Markov chain prior and exponential (*M* = 0.3, initial = 0.3) distribution were used for clock mean and standard deviation prior parameters, respectively. For each model, we conducted Monte Carlo Markov chains (MCMC) of 100 million generations, sampling every 10,000 steps. Runs were repeated to check for convergence and combined to obtain single model parameter estimates. Log parameters were inspected in Tracer v1.5 (http://beast.bio.ed.ac.uk/tracer), and burn‐ins of 10% were removed prior to combining runs. Statistical uncertainties in model parameter estimates are reported as 95% highest posterior distributions (HPD) around parameter means. Substitution models, tree priors, and clock models were selected via comparison of marginal likelihoods of combined runs, which were estimated using a stepping stone algorithm implemented within BEAST (Baele, Li, Drummond, Suchard, & Lemey, 2013; Baele et al., 2012).

Ancestral pathotypes and geographical origins were inferred in BEAST by reconstructing discrete traits onto the final rooted time‐measured MDV phylogeny (Lemey, Rambaut, Drummond, & Suchard, 2009) using a symmetric substitution model. MCMC were run and combined as described above, followed by mapping of inferred ancestral traits onto a maximum clade credibility (MCC) tree. Geographical locations (North America, Eurasia) were inferred from the literature as were pathotypes, which are based on a standardized in vivo virulence grading scheme (Witter, 1997), ranging from mild (m), virulent (v), very virulent (vv), and very virulent+ (vv+) categories. More recent field strains with a history of high virulence but not pathotyped within this grading scheme were treated as a separate category that we refer to here as “hypervirulent” (hv).

Mutations in ORFs MDV010‐MDV096 and R‐LORF4 were quantified manually following generation of individual ORF alignments with MAFFT and using the Md5 reference genome to guide gene annotations. Mutations recorded in MDV094‐96 did not include sample Md11 due to incomplete genomic data for this sample. For all genes, synonymous and nonsynonymous substitutions as well as indels were recorded. For comparisons of mutations across clades, point mutations from either tree tips or reconstructed internal nodes were used. Ancestral consensus sequences of the internal nodes EUA and NA were analyzed by separate pairwise comparison against the reconstructed tree root sequence. Ancestral consensus sequences for internal nodes of the MCC tree were reconstructed in FastML (Ashkenazy et al., 2012) with a GTR + Γ substitution model and marginal inference approach. Mutations were standardized (total number of mutations divided by ORF length—mutations/*L*) to give the average number of mutations per site within each ORF.

## 3 RESULTS

**Figure 1:**
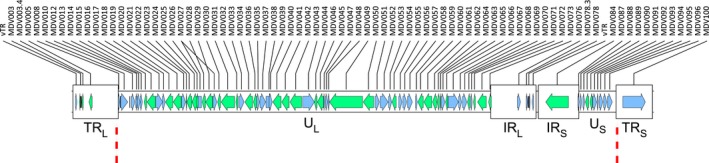
A scheme of the Marek's disease virus (MDV) genome. The MDV genome consists of one long (U_L_) and one short (U_S_) unique region separated by a variable long (IR_L_) and short (IR_S_) internal repeat region, in turn flanked on either side by a variable long (TR_L_) and short (TR_S_) terminal repeat regions. The segment analyzed in this study is indicated with dashed lines

The class E genomes of MDV consist of one long (U_L_) and one short (U_S_) unique region that are separated by a variable long (IR_L_) and short (IR_S_) internal repeat region (Figure 1). Together, this core portion of the genome contains all the predicted ORFs. There are additional inverted‐repeat long (TR_L_) and inverted‐short (TR_S_) regions that flank this core region. As the TR_L_ and TR_S_ are identical to the IR_L_ and IR_S_, respectively, those regions were not used in subsequent analysis. The final alignment used in phylogenetic analysis consisted of 141, 110 bp, including 643 variable sites, of which 353 were singletons. Variation was distributed unevenly across the alignment. The first half, coinciding approximately with the U_L_, proved to be highly conserved with variation steadily increasing thereafter and peaking in the repeat regions of the genome (IR_L_ and IR_S_) before decreasing again across the U_S_ (Fig. S1).

### 3.1 Evolution

**Figure 2:**
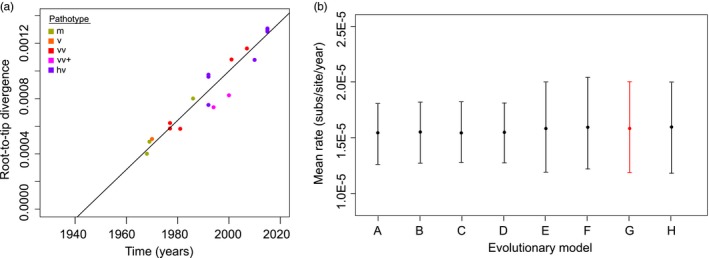
Evolutionary rate and temporal signal in the dataset. (a) Linear regression between maximum‐likelihood tree root‐to‐tip divergences and time: *R* ^2^ = 0.93; correlation coefficient = .97. Virulence pathotypes are color coded as follows: m = olive; v = orange; vv = red; vv+ = magenta; hv = purple. (b) Mean evolutionary rate for different evolutionary models, with bars indicating the 95% highest posterior distributions range. Model (substitution, clock, population size) A = HKY + Γ_4_, strict, constant; B = HKY + Γ_4,_ strict, exponential; C = GTR + Γ_4_, strict, constant; D = GTR + Γ_4_, strict, exponential; E = HKY + Γ_4_, relaxed, constant; F = HKY + Γ_4_, relaxed, exponential; G = GTR + Γ_4_, relaxed, constant; H = GTR + Γ_4_, relaxed, exponential. The selected model (G) is shown in red. HKY, Hasegawa, Kishino, and Yano; GTR, generalized time‐reversible; m, mild; v, virulent; vv, very virulent; vv+, very virulent+; hv, hypervirulent

Two genome sequences, GA and 584Ap80C (BAC20), were removed from the alignment after inspection of a linear regression between ML tree root‐to‐tip divergences and time. The passaging history of GA before sequencing is unknown and has nucleotide sequence discrepancies in 14 genes (S. Spatz, pers. comm.), while 584Ap80C has a history of 80 passages in CEC and is known to be attenuated (Schumacher et al., 2000). The disruptive influence of the two sequences on the temporal signal (but not topology) of the tree is therefore not surprising (Fig. S2a vs. b). An analysis of recombination in the remaining 20 MDV genomes pointed to the possible influence of recombination in the evolutionary history of MDV. Two events were detected in two neighboring regions of the alignment spanning ORFs MDV040 to MDV066 and MDV072 to MDV073, respectively (Table S2). The impact of the recombinant regions on ML tree topology was minimal, but their removal influenced temporal signal (Fig. S2b vs. c). The resulting temporal signal in the final dataset was high (Figure 2a; *R* ^2^ = 0.93; Correlation Coefficient = .97), indicating that a time‐scaled phylogenetic analysis was appropriate.

For dated phylogenetic reconstructions in BEAST, a GTR + Γ_4_ site model, constant size coalescent tree prior, and relaxed uncorrelated lognormal clock (Drummond, Ho, Phillips, & Rambaut, 2006) were used. These were selected following Bayes factor comparison of marginal‐likelihood estimates (Table 1). Selection of a relaxed clock was further supported by the distribution of the coefficient of rate variation, which fell between 0 and 1 and excluded zero (*M* = 0.204, 95% HPD = 0.023–0.376). However, we note that the deviation from a strict clock is not substantial for this dataset, as evidenced by the relatively low coefficient of rate variation, the difference in marginal likelihoods, and the similarity of evolutionary parameters that were estimated across models (Figure 2b). The HPD for growth rate did not exclude zero (*M* = −0.0004, 95% HPD = −0.033–0.032), indicating that a constant size coalescent tree prior was preferable to an exponential coalescent tree prior. The mean evolutionary rate for the selected evolutionary model was 1.58 × 10^−5^ substitutions per site per year (95% HPD values = 1.19 × 10^−5^–2.00 × 10^−5^). This was similar to rates inferred from a range of implemented models (Figure 2b) and is slightly higher than the rate of ~1 × 10^−5^ subs per site per year reported for a distantly related dsDNA virus *Myxoma virus* (MYXV, family Poxviridae) (Kerr et al., 2012, 2017).

**Table 1:** Model selection for phylogenetic analysis

Site model	Demographic model	Clock model	Marginal LnL	Preferred model^a^
GTR + Γ_4_	Exponential	Lognormal	−202,049.682	
HYK + Γ_4_	Exponential	Lognormal	−202,055.683	
GTR + Γ_4_	Exponential	Strict	−202,050.507	
HYK + Γ_4_	Exponential	Strict	−202,057.989	
GTR + Γ_4_	Constant	Lognormal	−202,048.025	*
HYK + Γ_4_	Constant	Lognormal	−202,054.499	
GTR + Γ_4_	Constant	Strict	−202,050.300	
HYK + Γ_4_	Constant	Strict	−202,057.217	

a Marginal log likelihoods (LnL) as inferred from stepping stone analysis for different combinations of substitution, clock, and population size models. *The evolutionary model was selected based on BF comparison of marginal LnLs (Kass & Raftery, 1995).

HKY, Hasegawa, Kishino, and Yano; GTR, generalized time‐reversible.

**Figure 3:**
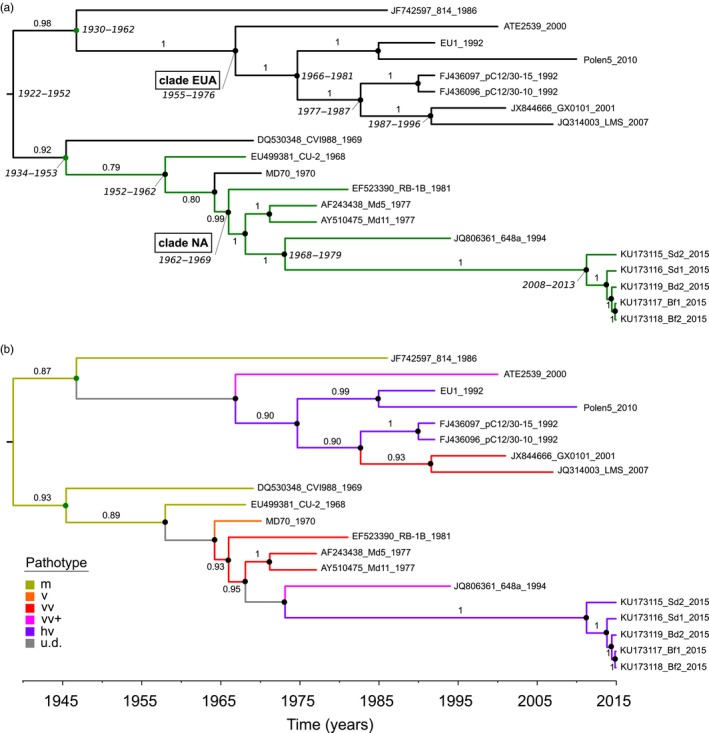
The time‐scaled Marek's disease virus phylogeny. Shown are maximum clade credibility (MCC) trees derived using the selected evolutionary model G. Node support is given as posterior probabilities, represented by a black (>.99) or red (>.85) circle. Posterior support (>.7) for ancestral reconstructions at internal parts of the tree is indicated above individual branches, which are colored according to the corresponding trait reconstruction. Reconstructions with low support (<.7) are indicated in gray. (a) MCC tree overlaid with ancestral geographical reconstruction. Black = Eurasian, green = North American. The 95% highest posterior distributions range for tMRCAs (in years) is indicated by a line for relevant nodes. (b) MCC tree overlaid with ancestral pathotype reconstruction, with the color‐coding following Figure 2

The inferred root of the time‐scaled phylogeny of MDV is located at a point that is equidistant from the two furthest tips of the ML tree (midpoint) (Fig. S2c), affirming the close correlation between virus sampling date and root‐to‐tip evolutionary divergence, and the moderately clock‐like evolution of MDV over time. The MCC tree indicates the presence of geographical structuring of MDV strains, with the reconstruction supporting a clade of virulent North American viruses emerging from within a clade of mild (m) or virulent (v) strains of Eurasian or North American descent. All other viruses constitute what appears to be a single Eurasian clade, with ancestral reconstructions also indicating a Eurasian origin at the root of the MDV tree (Figure 3a). The time to most recent common ancestor (tMRCA) of the sampled MDV genomes (*M* = 1938, 95% HPD = 1922–1952) precedes the earliest records of increasing disease severity in reared poultry by approximately a decade (Benton & Cover, 1957). More virulent viruses fall further from the root of the tree (Figure 2a) and occupy derived positions within Eurasian and North American clades (Figure 3b). Our reconstruction of ancestral pathotypes of the sampled MDV viruses suggests that MDV virulence evolved independently: at least once in Eurasia and in North America. Interestingly, virulent viruses (vv, vv+ or hv) are estimated to have evolved in Eurasia (termed clade EUA) and North America (termed clade NA) at approximately the same time, with the following tMRCAs: NA *M* = 1966 (95% HPD = 1962–1969); EUA *M* = 1966 (95% HPD = 1955–1976). It is noteworthy that these estimates lie close to the year in which comprehensive vaccination programs were introduced both in the United States and in Europe.

### 3.2 Mutation analysis

**Figure 4:**
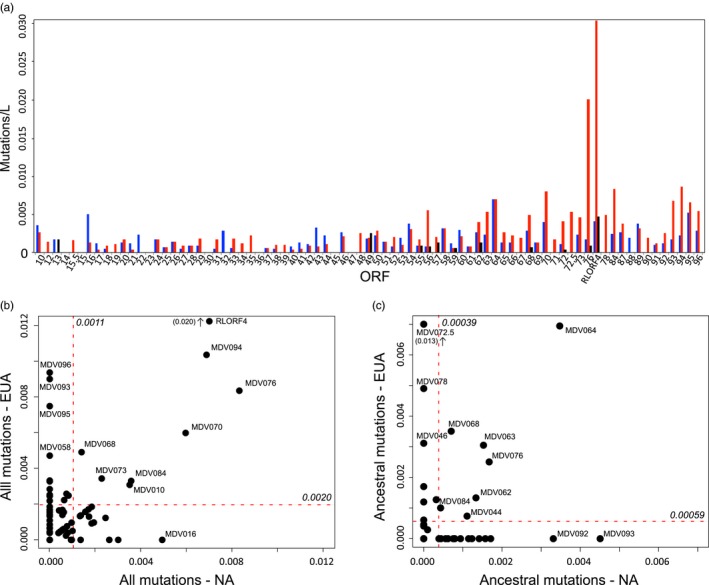
Mutations in open‐reading frames (ORF)s across the alignment and in clades EUA/NA. (a) Total number of standardized mutations for all samples (total number of mutations divided by ORF length), to give the average number of mutations per site for each ORF. Red = nonsynonymous mutations, blue = synonymous mutations, black = indels. (b) Comparison of standardized point mutations in virulent Eurasian (EUA) and North American (NA) strains only. (c) Comparison of point mutations in the reconstructed ancestor at nodes EUA and NA as compared against the root of the tree. The mean number of mutations per site per clade is indicated with a red line in both panels

Variation in standardized ORF mutations mirrored the pattern of genetic variation across the MDV genome with the majority of mutations falling in ORFs in the latter half of the U_L_, the U_S_, or the internal repeat regions (IR_L_, IR_S_) (Figure 4a, Fig. S1). We then looked beyond this broader pattern to search for genetic variation that may be associated with the evolution of MDV virulence. We compared standardized point mutations in contemporary strains within the virulent clades EUA and NA (Figure 4b), in addition to quantifying independent mutational changes that occurred between the root of the tree and either the MRCA of EUA or NA (Figure 4c, File S1). We did not detect a strong correlation in the pattern of ORF point mutations between clades EUA and NA in tip genetic variation (Spearman's *rho* = .205, *p*‐value = .069, Figure 4b), but a marginally significant correlation was detected in mutational changes that evolved between the root and the ancestral sequences of clades EUA and NA, respectively (Spearman's *rho* = .228, *p*‐value = .043, Figure 4c). Most ORFs harbored mutations that were not obviously associated across virulent viruses. However, we did identify a small number of ORFs with greater‐than‐average mutations in both EUA and NA (Table 2). Notably, Meq (MDV076), ICP4 (MDV084), and ICP27 (MDV068), which all function as general gene transactivators, were identified in the tip genetic variant analysis and also in ancestral reconstruction comparisons of the EUA and NA clades. In one case, a putative decrease in virulence was also predicted for a Eurasian lineage of viruses (GX0101 and LMS) (Figure 3), which is associated with substitutions in the above genes, including modifications to specific nonsynonymous nucleotide sites in ICP27 and Meq (Fig. S3, File S1).

**Table 2:** List of candidate genes associated with the evolution of Marek's disease virus (MDV) virulence

Locus	Other names	Description	EUA‐NA all^a^	EUA‐NA ancestor^b^
MDV076	Meq, Eco Q	Oncoprotein, DNA‐binding transcription factor related to bZIP proteins, binds to CtBP	*	*
MDV084	RS1, ICP4	Immediate‐early gene transactivator, ICP4‐like protein, migrates to nucleus to bind DNA transactivating viral genes	*	*
MDV068	UL54, ICP27	Multifunctional expression regulator, RNA‐binding protein, exports virus mRNA from nucleus	*	*
R‐LORF4		Contains a potential transmembrane domain	*	
MDV094	US6	Membrane glycoprotein D‐like protein, contains a signal peptide, binds cell‐surface receptors	*	
MDV070	UL55‐like	Nuclear protein	*	
MDV073	pp38	38kD phosphoprotein	*	
MDV010	Viral lipase	Virulence factor, contains a signal peptide, glycoprotein, probably not enzymatically active	*	
MDV064	UL49.5, gN	Envelope glycoprotein (type 1), modulates membrane fusion activity, crucial role in virion morphogenesis, complexes with gM		*
MDV063	UL50	Deoxyuridine triphosphatase (dUTPase)‐like protein		*
MDV062	UL49, VP22	Tegument protein, role in accumulation of viral mRNA and translation during late infection		*
MDV044	UL31	Nuclear phosphoprotein‐like protein, interacts with nuclear egress membrane protein		*

a Based on greated‐than‐average number of point mutations found in contemporary strains of both EUA and NA.

b Based on greated‐than‐average number of point mutations found in the reconstructed ancestors of both EUA and NA (compared to the root of the tree).

## 4 DISCUSSION

Understanding the evolutionary history of MDV is important for two reasons. Firstly, the history of MDV in the 20th century represents a very useful case study for understanding the evolutionary origin and causes of increasing virulence. Here, high‐throughput sequencing in combination with a novel MDV tiling enrichment array has enabled us to determine genomic sequences quickly and gain new insight into the genomic origins and underlying mechanisms of this process. Secondly, understanding how and why MDV virulence has increased is of significant applied relevance not just for the poultry industry, but also in the wider context of the emergence of treatment‐resistant pathogens. The increasing incidence of drug and vaccine resistance represents a major global challenge to human and animal health, companion animals, and wildlife alike, yet meaningful solutions to this growing problem are lacking.

The paucity of knowledge concerning the evolution of dsDNA viruses is particularly surprising given their medical and veterinary importance. Our use of MDV samples collected between 1968 and 2015 enables a first insight into the origin and tempo of MDV evolution, representing the first alphaherpesvirus affecting agricultural animals to have undergone such a detailed genome‐scale phylogenetic analysis. Our temporal phylogeny of MDV yielded an evolutionary rate of ~1.6 × 10^−5^, which is in line with a range of rates that have been recorded for similarly sized dsDNA viruses such as variola virus (~9 × 10^−6^) (Duggan et al., 2016; Firth et al., 2010) and MYXV (~1 × 10^−5^) (Kerr et al., 2012, 2017). Overall, such rates are generally higher than typically expected for dsDNA viruses and imply higher rates of nucleotide substitution than those inferred by host–virus codivergence analysis (Firth et al., 2010). One possibility is that these viruses have undergone sustained selective pressure, which could have affected the observable substitution rate. For MDV, this would indeed be expected given a recent history of widespread vaccine use and intensive farming practices, which significantly affect MDV replication and transmission. Indeed, of all point mutations detected in the screened ORFs, over 62% were nonsynonymous, implying a role for positive selection during the evolution of MDV (Padhi & Parcells, 2016). Unfortunately, the low number of variable positions across ORFs prevented formal tests of gene or site‐specific selection in the current study.

An examination of point mutations in the ORFs of virulent strains failed to identify a strong correlation between substitution patterns in virulent viruses, suggesting that multiple genotypic pathways underlie the evolution of MDV virulence in Eurasia and North America. Despite this general pattern, we did identify a minority of candidate ORFs with mutations that appeared to be associated specifically with virulent MDV strains (Table 2) including the general transactivators Meq (MDV076), ICP4 (MDV084), and ICP27 (MDV068). Several of the listed genes have been shown experimentally to play a role in MDV pathogenicity (Amor et al., 2011; Brown et al., 2006; Jarosinski, Osterrieder, Nair, & Schat, 2005; Kamil et al., 2005; Liu et al., 1999; Lupiani et al., 2004; Nair, 2013; Reddy et al., 2002; Tischer, Schumacher, Messerle, Wagner, & Osterrieder, 2002). While known as an important MDV oncoprotein, Meq is also expressed during lytic replication and has a potential role in the rapid production of virus progeny (Coupeau, Dambrine, & Rasschaert, 2012). The genomic region flanking Meq and the latency‐associated transcript region on the antisense strand immediately downstream of ICP4 are known to harbor a number of microRNAs on the antisense strand (Burnside et al., 2006; Morgan & Burnside, 2011; Xu et al., 2008; Yao, Zhao, Smith, Watson, & Nair, 2009), which in the context of MDV virulence evolution should not be overlooked (Zhao et al., 2011). Another particularly variable ORF associated with virulent Eurasian and North American viruses is R‐LORF4. R‐LORF4 is in the direct vicinity of Meq, and splice variants between Meq and R‐LORF4 have been identified. In addition, R‐LORF4 was shown to be a virulence factor, much like Meq (Jarosinski et al., 2005; Kim, Hunt, & Cheng, 2010). However, overall we consider it as likely that subtle epistatic interactions among many genes and noncoding regions, which are difficult to characterize and detect, have also played a significant role in MDV virulence evolution. For example, of the genes associated with virulence, only 10% were shared by both virulent Eurasian and North American strains, with the majority of genes either containing no variation (>50%) or harboring variants that were specific to one but not both lineages (10%–20%). This is not surprising given the size and complexity of the MDV genome, and it supports findings from recent studies of virulence evolution in the distantly related dsDNA virus MYXV, where similar patterns of genotypic evolution were observed during the processes of attenuation and virulence adaptation (Kerr et al., 2012, 2017). This contrasts with smaller RNA viruses such as HIV‐1 and influenza (Baigent & McCauley, 2003; Kimata, Kuller, Anderson, Dailey, & Overbaugh, 1999), where the limited repertoire of genes is thought to restrict the mutational landscape and therefore the available routes to adaptive evolution.

The root of the MDV phylogeny is predicted to precede the first records of increased MDV infection severity by only a decade or so, indicating that the diversity of MDV sampled globally since the 1960s has a recent evolutionary past. MDV was first described by Jozsef Marek in Hungary, 1907 (Marek, 1907), but the wider history of MDV in Eurasia or North America is not clearly understood. It is possible that the virus and the chicken host have undergone a long coevolutionary history (Weiss & Biggs, 1972), in which case our analysis indicates expansion of a relatively narrow subset of MDV in the 20th century. This is perhaps not surprising in the context of increasingly uniform selection dynamics: For example, most production broiler lines have significantly introgressed B21 (MHC‐I) alleles. Further whole‐genome sampling from additional geographical sources and historical periods, particularly prior to the 1960s, is required to strengthen support for the inferred phylogeography and patterns of historical MDV virulence presented here. It may also be useful in future to sample feral junglefowl and related species, which although less commonly infected (Nair, 2005), will help to characterize the wider genetic diversity of MDV, particularly because such wild bird populations have not been exposed to the extreme and artificial selective pressures found on poultry farms.

An interesting aspect of our genome‐scale analysis is that MDV virulence appears to have evolved independently in Eurasia and North America, respectively. Virulent strains clustered together in derived positions within well‐supported and geographically coherent clades. The ancestors of these clades are predicted to have emerged in concert with or soon after the introduction of vaccines to control clinical disease induced by MDV, which had been increasing in frequency and severity in the late 1950s and 1960s (Witter, 1997). In this context, it is of interest to note that two different routes to vaccine development were followed in Europe and in North America. HPRS‐16, originally isolated and characterized in the UK, is an MDV strain that exhibited lower virulence and was modified by serial passage to a homotypic modified‐live virus vaccine (Churchill, Payne, & Chubb, 1969). On the other hand, HVT was developed as a heterotypic vaccine in the USA (Okazaki, Purchase, & Burmester, 1970). Both vaccines were developed simultaneously for use in Europe and North America, respectively, very shortly after the agent responsible for disease was first identified (Churchill & Biggs, 1967; Purchase & Biggs, 1967). Whereas in the USA, bivalent vaccines were subsequently adopted in the early 1980s in the wake of HVT vaccine failures, in Europe a vaccine derived from a naturally mild isolate of MDV (Rispens/CVI988) was used and later disseminated globally in the 1990s. Given the different approaches of vaccinal control of MDV in different regions of the world, it is not entirely surprising that two clearly distinguishable routes to vaccine resistance may have evolved in each continent over the past five decades.

## DATA ARCHIVING STATEMENT

Genome sequences are available on GenBank under the following accession numbers: MF431493‐6. All other raw data including alignments, phylogenetic trees, and mutation information across ORFs are available at the Dryad Digital repository: https://doi.org/10.5061/dryad.mq2q9.

## References

[eva12515-bib-0001] Alizon, S. , Hurford, A. , Mideo, N. , & Van Baalen, M. (2009). Virulence evolution and the trade‐off hypothesis: History, current state of affairs and the future. Journal of Evolutionary Biology, 22, 245–259. 19196383 10.1111/j.1420-9101.2008.01658.x

[eva12515-bib-0002] Amor, S. , Strassheim, S. , Dambrine, G. , Remy, S. , Rasschaert, D. , & Laurent, S. (2011). ICP27 protein of Marek's disease virus interacts with SR proteins and inhibits the splicing of cellular telomerase chTERT and viral vIL8 transcripts. Journal of General Virology, 92, 1273–1278. 21325479 10.1099/vir.0.028969-0

[eva12515-bib-0003] Anderson, R. M. , & May, R. M. (1982). Coevolution of hosts and parasites. Parasitology, 85, 411–426. 6755367 10.1017/s0031182000055360

[eva12515-bib-0004] Andrews, S. (2010). FastQC: A quality control tool for high throughput sequence data. Retrieved from http://www.bioinformatics.babraham.ac.uk/projects/fastqc

[eva12515-bib-0005] Anthony, N. B. (1998). A review of genetic practices in poultry: Efforts to improve meat quality. Journal of Muscle Foods, 9, 25–33.

[eva12515-bib-0006] Ashkenazy, H. , Penn, O. , Doron‐Faigenboim, A. , Cohen, O. , Cannarozzi, G. , Zomer, O. , & Pupko, T. (2012). FastML: A web server for probabilistic reconstruction of ancestral sequences. Nucleic Acids Research, 40, W580–W584. 22661579 10.1093/nar/gks498 PMC3394241

[eva12515-bib-0007] Atkins, K. E. , Read, A. F. , Savill, N. J. , Renz, K. G. , Fakhrul Islam, A. F. M. , Walkden‐Brown, S. W. , & Woolhouse, M. E. (2013). Vaccination and reduced cohort duration can drive virulence evolution: Marek's disease virus and intensified agriculture. Evolution, 67, 851–860. 23461333 10.1111/j.1558-5646.2012.01803.x

[eva12515-bib-0008] Atkins, K. E. , Read, A. F. , Walkden‐Brown, S. W. , Savill, N. J. , & Woolhouse, M. E. J. (2013). The effectiveness of mass vaccination on Marek's disease virus (MDV) outbreaks and detection within a broiler barn: A modeling study. Epidemics, 5, 208–217. 24267877 10.1016/j.epidem.2013.10.001 PMC3863959

[eva12515-bib-0009] Baele, G. , Lemey, P. , Bedford, T. , Rambaut, A. , Suchard, M. A. , & Alekseyenko, A. V. (2012). Improving the accuracy of demographic and molecular clock model comparison while accommodating phylogenetic uncertainty. Molecular Biology and Evolution, 29, 2157–2167. 22403239 10.1093/molbev/mss084 PMC3424409

[eva12515-bib-0010] Baele, G. , Li, W. L. S. , Drummond, A. J. , Suchard, M. A. , & Lemey, P. (2013). Accurate model selection of relaxed molecular clocks in Bayesian phylogenetics. Molecular Biology and Evolution, 30, 239–243. 23090976 10.1093/molbev/mss243 PMC3548314

[eva12515-bib-0011] Baigent, S. J. , & McCauley, J. W. (2003). Influenza type A in humans, mammals and birds: Determinants of virus virulence, host‐range and interspecies transmission. Bioessays, 25, 657–671. 12815721 10.1002/bies.10303

[eva12515-bib-0012] Benton, W. J. , & Cover, M. S. (1957). The increased incidence of visceral lymphomatosis in broiler and replacement birds. Avian Diseases, 1, 320–327.

[eva12515-bib-0013] Bolger, A. M. , Lohse, M. , & Usadel, B. (2014). Trimmomatic: A flexible trimmer for illumina sequence data. Bioinformatics, 30, 2114–2120. 24695404 10.1093/bioinformatics/btu170 PMC4103590

[eva12515-bib-0014] Brown, A. C. , Baigent, S. J. , Smith, L. P. , Chattoo, J. P. , Petherbridge, L. J. , Hawes, P. , … Nair, V. (2006). Interaction of MEQ protein and C‐terminal‐binding protein is critical for induction of lymphomas by Marek's disease virus. Proceedings of the National Academy of Sciences of the United States of America, 103, 1687–1692. 16446447 10.1073/pnas.0507595103 PMC1413633

[eva12515-bib-0015] Bull, J. J. , & Lauring, A. S. (2014). Theory and empiricism in virulence evolution. PLoS Pathogens, 10, e1004387. 25340792 10.1371/journal.ppat.1004387 PMC4207818

[eva12515-bib-0016] Burnside, J. , Bernberg, E. , Anderson, A. , Lu, C. , Meyers, B. C. , Green, P. J. , … Morgan, R. W. (2006). Marek's disease virus encodes microRNAs that map to meq and the latency‐associated transcript. Journal of Virology, 80, 8778–8786. 16912324 10.1128/JVI.00831-06 PMC1563840

[eva12515-bib-0017] Carroll, S. P. , Jorgensen, P. S. , Kinnison, M. T. , Bergstrom, C. T. , Denison, R. F. , Gluckman, P. , … Tabashnik, B. E. (2014). Applying evolutionary biology to address global challenges. Science, 346, 1245993. 25213376 10.1126/science.1245993 PMC4245030

[eva12515-bib-0018] Castresana, J. (2000). Selection of conserved blocks from multiple alignments for their use in phylogenetic analysis. Molecular Biology and Evolution, 17, 540–552. 10742046 10.1093/oxfordjournals.molbev.a026334

[eva12515-bib-0019] Churchill, A. E. , & Biggs, P. M. (1967). Agent of Marek's disease in tissue culture. Nature, 215, 528–530. 4293679 10.1038/215528a0

[eva12515-bib-0020] Churchill, A. E. , Payne, L. N. , & Chubb, R. C. (1969). Immunization against Marek's disease using a live attenuated virus. Nature, 221, 744–747. 4304053 10.1038/221744a0

[eva12515-bib-0021] Coupeau, D. , Dambrine, G. , & Rasschaert, D. (2012). Kinetic expression analysis of the cluster mdv1‐mir‐M9–M4, genes meq and vIL‐8 differs between the lytic and latent phases of Marek's disease virus infection. Journal of General Virology, 93, 1519–1529. 22442112 10.1099/vir.0.040741-0

[eva12515-bib-0022] Cressler, C. E. , McLeod, D. V. , Rozins, C. , van den Hoogen, J. , & Day, T. (2016). The adaptive evolution of virulence: A review of theoretical predictions and empirical tests. Parasitology, 143, 915–930. 26302775 10.1017/S003118201500092X PMC4873896

[eva12515-bib-0023] Daszak, P. (2000). Emerging infectious diseases of wildlife – Threats to biodiversity and human health (vol 287, pg 443, 2000). Science, 287, 1756–1756. 10.1126/science.287.5452.443 10642539

[eva12515-bib-0024] Drummond, A. J. , Ho, S. Y. , Phillips, M. J. , & Rambaut, A. (2006). Relaxed phylogenetics and dating with confidence. PLoS Biology, 4, e88. 16683862 10.1371/journal.pbio.0040088 PMC1395354

[eva12515-bib-0025] Drummond, A. J. , Suchard, M. A. , Xie, D. , & Rambaut, A. (2012). Bayesian phylogenetics with BEAUti and the BEAST 1.7. Molecular Biology and Evolution, 29, 1969–1973. 22367748 10.1093/molbev/mss075 PMC3408070

[eva12515-bib-0026] Duggan, A. T. , Perdomo, M. F. , Piombino‐Mascali, D. , Marciniak, S. , Poinar, D. , Emery, M. V. , … Poinar, H. N. (2016). 17th century variola virus reveals the recent history of smallpox. Current Biology, 26, 3407–3412. 27939314 10.1016/j.cub.2016.10.061 PMC5196022

[eva12515-bib-0027] Ebert, D. (1998). Evolution – Experimental evolution of parasites. Science, 282, 1432–1435. 9822369 10.1126/science.282.5393.1432

[eva12515-bib-0028] Ebert, D. , & Bull, J. J. (2003). Challenging the trade‐off model for the evolution of virulence: Is virulence management feasible? Trends in Microbiology, 11, 15–20. 12526850 10.1016/s0966-842x(02)00003-3

[eva12515-bib-0029] Ewald, P. W. (1983). Host‐parasite relations, vectors, and the evolution of disease severity. Annual Review of Ecology and Systematics, 14, 465–485.

[eva12515-bib-0030] Firth, C. , Kitchen, A. , Shapiro, B. , Suchard, M. A. , Holmes, E. C. , & Rambaut, A. (2010). Using time‐structured data to estimate evolutionary rates of double‐stranded DNA viruses. Molecular Biology and Evolution, 27, 2038–2051. 20363828 10.1093/molbev/msq088 PMC3107591

[eva12515-bib-0031] Gandon, S. , Mackinnon, M. J. , Nee, S. , & Read, A. F. (2001). Imperfect vaccines and the evolution of pathogen virulence. Nature, 414, 751–756. 11742400 10.1038/414751a

[eva12515-bib-0032] Garrison, E. , & Marth, G. (2012). Haplotype‐based variant detection from short‐read sequencing. ArXiv:1207.3907v2.

[eva12515-bib-0033] Guindon, S. , Dufayard, J. F. , Lefort, V. , Anisimova, M. , Hordijk, W. , & Gascuel, O. (2010). New algorithms and methods to estimate maximum‐likelihood phylogenies: Assessing the performance of PhyML 3.0. Systematic Biology, 59, 307–321. 20525638 10.1093/sysbio/syq010

[eva12515-bib-0034] Hasegawa, M. , Kishino, H. , & Yano, T. (1985). Dating of the human‐ape splitting by a molecular clock of mitochondrial DNA. Journal of Molecular Evolution, 22, 160–174. 3934395 10.1007/BF02101694

[eva12515-bib-0035] Hawley, D. M. , Osnas, E. E. , Dobson, A. P. , Hochachka, W. M. , Ley, D. H. , & Dhondt, A. A. (2013). Parallel patterns of increased virulence in a recently emerged wildlife pathogen. PLoS Biology, 11, e1001570. 23723736 10.1371/journal.pbio.1001570 PMC3665845

[eva12515-bib-0036] Jarosinski, K. W. , Osterrieder, N. , Nair, V. K. , & Schat, K. A. (2005). Attenuation of Marek's disease virus by deletion of open reading frame RLORF4 but not RLORF5a. Journal of Virology, 79, 11647–11659. 16140742 10.1128/JVI.79.18.11647-11659.2005 PMC1212595

[eva12515-bib-0037] Kamil, J. P. , Tischer, B. K. , Trapp, S. , Nair, V. K. , Osterrieder, N. , & Kung, H. J. (2005). vLIP, a viral lipase homologue, is a virulence factor of Marek's disease virus. Journal of Virology, 79, 6984–6996. 15890938 10.1128/JVI.79.11.6984-6996.2005 PMC1112136

[eva12515-bib-0038] Kass, R. E. , & Raftery, A. E. (1995). Bayes factors. Journal of American Statistical Association, 90, 773–795.

[eva12515-bib-0039] Katoh, K. , & Standley, D. M. (2013). MAFFT multiple sequence alignment software version 7: Improvements in performance and usability. Molecular Biology and Evolution, 30, 772–780. 23329690 10.1093/molbev/mst010 PMC3603318

[eva12515-bib-0040] Kerr, P. J. , Cattadori, I. M. , Rogers, M. B. , Fitch, A. , Geber, A. , Liu, J. , … Holmes, E. C. (2017). Genomic and phenotypic characterization of myxoma virus from Great Britain reveals multiple evolutionary pathways distinct from those in Australia. PLoS Pathogens, 13, e1006252. 28253375 10.1371/journal.ppat.1006252 PMC5349684

[eva12515-bib-0041] Kerr, P. J. , Ghedin, E. , DePasse, J. V. , Fitch, A. , Cattadori, I. M. , Hudson, P. J. , … Holmes, E. C. (2012). Evolutionary history and attenuation of myxoma virus on two continents. PLoS Pathogens, 8, e1002950. 23055928 10.1371/journal.ppat.1002950 PMC3464225

[eva12515-bib-0042] Kim, T. , Hunt, H. D. , & Cheng, H. H. (2010). Marek's disease viruses lacking either R‐LORF10 or LORF4 have altered virulence in chickens. Virus Genes, 40, 410–420. 20229182 10.1007/s11262-010-0469-4

[eva12515-bib-0043] Kimata, J. T. , Kuller, L. , Anderson, D. B. , Dailey, P. , & Overbaugh, J. (1999). Emerging cytopathic and antigenic simian immunodeficiency virus variants influence AIDS progression. Nature Medicine, 5, 535–541. 10.1038/8414 10229230

[eva12515-bib-0044] Lemey, P. , Rambaut, A. , Drummond, A. J. , & Suchard, M. A. (2009). Bayesian phylogeography finds its roots. PLoS Computational Biology, 5, e1000520. 19779555 10.1371/journal.pcbi.1000520 PMC2740835

[eva12515-bib-0045] Li, H. , & Durbin, R. (2009). Fast and accurate short read alignment with Burrows‐Wheeler transform. Bioinformatics, 25, 1754–1760. 19451168 10.1093/bioinformatics/btp324 PMC2705234

[eva12515-bib-0046] Li, H. , Handsaker, B. , Wysoker, A. , Fennell, T. , Ruan, J. , Homer, N. , … Durbin, R. (2009). The sequence alignment/map format and SAMtools. Bioinformatics, 25, 2078–2079. 19505943 10.1093/bioinformatics/btp352 PMC2723002

[eva12515-bib-0047] Liu, J. L. , Ye, Y. , Qian, Z. , Qian, Y. , Templeton, D. J. , Lee, L. F. , & Kung, H. J. (1999). Functional interactions between herpesvirus oncoprotein MEQ and cell cycle regulator CDK2. Journal of Virology, 73, 4208–4219. 10196317 10.1128/jvi.73.5.4208-4219.1999 PMC104200

[eva12515-bib-0048] Lupiani, B. , Lee, L. F. , Cui, X. , Gimeno, I. , Anderson, A. , Morgan, R. W. , … Reddy, S. M. (2004). Marek's disease virus‐encoded Meq gene is involved in transformation of lymphocytes but is dispensable for replication. Proceedings of the National Academy of Sciences of the United States of America, 101, 11815–11820. 15289599 10.1073/pnas.0404508101 PMC511057

[eva12515-bib-0049] Marek, J. (1907). Multiple nervenentzündung (polyneuritis) bei hühnern. Deutsche Tierärztliche Wochenschrift, 15, 417–421.

[eva12515-bib-0050] Martin, D.P. , Murrell, B. , Golden, M. , Khoosal, A. , & Muhire, B. (2015). RDP4: Detection and analysis of recombination patterns in virus genomes. Virus Evolution, 1, vev003. 27774277 10.1093/ve/vev003 PMC5014473

[eva12515-bib-0051] Morens, D. M. , Folkers, G. K. , & Fauci, A. S. (2004). The challenge of emerging and re‐emerging infectious diseases. Nature, 430, 242–249. 15241422 10.1038/nature02759 PMC7094993

[eva12515-bib-0052] Morgan, R.W. , & Burnside, J. (2011). Roles of avian herpesvirus microRNAs in infection, latency, and oncogenesis. Biochimica et Biophysica Acta (BBA) ‐ Gene Regulatory Mechanisms, 1809, 654–659. 21683170 10.1016/j.bbagrm.2011.06.001

[eva12515-bib-0053] Morse, S. S. , Mazet, J. A. K. , Woolhouse, M. , Parrish, C. R. , Carroll, D. , Karesh, W. B. , … Daszak, P. (2012). Zoonoses 3 prediction and prevention of the next pandemic zoonosis. Lancet, 380, 1956–1965. 23200504 10.1016/S0140-6736(12)61684-5 PMC3712877

[eva12515-bib-0054] Nair, V. (2005). Evolution of Marek's disease – A paradigm for incessant race between the pathogen and the host. The Veterinary Journal, 170, 175–183. 16129338 10.1016/j.tvjl.2004.05.009

[eva12515-bib-0055] Nair, V. (2013). Latency and tumorigenesis in Marek's disease. Avian Diseases, 57, 360–365. 23901747 10.1637/10470-121712-Reg.1

[eva12515-bib-0056] Okazaki, W. , Purchase, H. G. , & Burmester, B. R. (1970). Protection against Marek's disease by vaccination with a herpesvirus of turkeys. Avian Diseases, 14, 413–429. 4913403

[eva12515-bib-0057] Osterrieder, N. , Kamil, J. P. , Schumacher, D. , Tischer, B. K. , & Trapp, S. (2006). Marek's disease virus: From miasma to model. Nature Reviews Microbiology, 4, 283–294. 16541136 10.1038/nrmicro1382

[eva12515-bib-0058] Padhi, A. , & Parcells, M. S. (2016). Positive selection drives rapid evolution of the meq oncogene of Marek's disease virus. PLoS One, 11, e0162180. 27662574 10.1371/journal.pone.0162180 PMC5035050

[eva12515-bib-0059] Pfeifer, B. , Wittelsburger, U. , Ramos‐Onsins, S. E. , & Lercher, M. J. (2014). PopGenome: An efficient swiss army knife for population genomic analyses in R. Molecular Biology and Evolution, 31, 1929–1936. 24739305 10.1093/molbev/msu136 PMC4069620

[eva12515-bib-0060] Purchase, H. G. , & Biggs, P. M. (1967). Characterization of five isolates of Marek's disease. Research in Veterinary Science, 8, 440–449. 4294256

[eva12515-bib-0061] R Core Team (2016). R: A language and environment for statistical computing. Vienna, Austria: R Foundation for Statistical Computing.

[eva12515-bib-0062] Rambaut, A. , Lam, T. T. , Max Carvalho, L. , & Pybus, O. G. (2016). Exploring the temporal structure of heterochronous sequences using TempEst (formerly Path‐O‐Gen). Virus evolution, 2, vew007. 27774300 10.1093/ve/vew007 PMC4989882

[eva12515-bib-0063] Read, A. F. , Baigent, S. J. , Powers, C. , Kgosana, L. B. , Blackwell, L. , Smith, L. P. , … Nair, V. K. (2015). Imperfect vaccination can enhance the transmission of highly virulent pathogens. PLoS Biology, 13, e1002198. 26214839 10.1371/journal.pbio.1002198 PMC4516275

[eva12515-bib-0064] Reddy, S. M. , Lupiani, B. , Gimeno, I. M. , Silva, R. F. , Lee, L. F. , & Witter, R. L. (2002). Rescue of a pathogenic Marek's disease virus with overlapping cosmid DNAs: Use of a pp38 mutant to validate the technology for the study of gene function. Proceedings of the National Academy of Sciences of the United States of America, 99, 7054–7059. 11997455 10.1073/pnas.092152699 PMC124527

[eva12515-bib-0065] Rozins, C. , & Day, T. (2017). The industrialization of farming may be driving virulence evolution. Evolutionary Applications, 10, 189–198. 28127395 10.1111/eva.12442 PMC5253429

[eva12515-bib-0066] Sambrook, J. , Fritsch, E. F. , & Maniatis, T. (1989). Molecular cloning: A laboratory manual (2nd ed.). Cold Spring Harbor, NY: Cold Spring Harbor Laboratory Press.

[eva12515-bib-0067] Schmid‐Hempel, P. (2008). Parasite immune evasion: A momentous molecular war. Trends in Ecology & Evolution, 23, 318–326. 18439709 10.1016/j.tree.2008.02.011

[eva12515-bib-0068] Schmid‐Hempel, P. (2009). Immune defence, parasite evasion strategies and their relevance for ‘macroscopic phenomena’ such as virulence. Philosophical Transactions of the Royal Society of London B: Biological Sciences, 364, 85–98. 18930879 10.1098/rstb.2008.0157 PMC2666695

[eva12515-bib-0069] Schmid‐Hempel, P. (2011). *Evolutionary Parasitology: The integrated study of infections, immunology, ecology, and genetics* Oxford: Oxford University Press.

[eva12515-bib-0070] Schumacher, D. , Tischer, B. K. , Fuchs, W. , & Osterrieder, N. (2000). Reconstitution of Marek's disease virus serotype 1 (MDV‐1) from DNA cloned as a bacterial artificial chromosome and characterization of a glycoprotein B‐negative MDV‐1 mutant. Journal of Virology, 74, 11088–11098. 11070004 10.1128/jvi.74.23.11088-11098.2000 PMC113189

[eva12515-bib-0071] Smith, D. R. , & Mideo, N. (2017). Modelling the evolution of HIV‐1 virulence in response to imperfect therapy and prophylaxis. Evolutionary Applications, 10, 297–309. 28250813 10.1111/eva.12458 PMC5322411

[eva12515-bib-0072] Tamura, K. , Peterson, D. , Peterson, N. , Stecher, G. , Nei, M. , & Kumar, S. (2011). MEGA5: Molecular evolutionary genetics analysis using maximum likelihood, evolutionary distance, and maximum parsimony methods. Molecular Biology and Evolution, 28, 2731–2739. 21546353 10.1093/molbev/msr121 PMC3203626

[eva12515-bib-0073] Tischer, B. K. , Schumacher, D. , Messerle, M. , Wagner, M. , & Osterrieder, N. (2002). The products of the UL10 (gM) and the UL49.5 genes of Marek's disease virus serotype 1 are essential for virus growth in cultured cells. Journal of General Virology, 83, 997–1003. 11961253 10.1099/0022-1317-83-5-997

[eva12515-bib-0074] Tulman, E. R. , Afonso, C. L. , Lu, Z. , Zsak, L. , Rock, D. L. , & Kutish, G. F. (2000). The genome of a very virulent Marek's disease virus. Journal of Virology, 74, 7980–7988. 10933706 10.1128/jvi.74.17.7980-7988.2000 PMC112329

[eva12515-bib-0075] Weiss, R. A. , & Biggs, P. M. (1972). Leukosis and Marek's disease viruses of feral red jungle flow and domestic fowl in Malaya. Journal of the National Cancer Institute, 49, 1713–1725. 4119166 10.1093/jnci/49.6.1713

[eva12515-bib-0076] Witter, R. L. (1997). Increased virulence of Marek's disease virus field isolates. Avian Diseases, 41, 149–163. 9087332

[eva12515-bib-0077] Witter, R. L. (1998). The changing landscape of Marek's disease. Avian Pathology, 27, S46–S53.

[eva12515-bib-0078] Witter, R.L. (2001). Marek's disease vaccines – Past, present and future – [chicken vs virus – A battle of the centuries]. The Bart Rispens memorial lecture In Schat K. A., Morgan R. M., Parcells M. S. & Spencer J. L. (Eds.), Current progress on Marek's disease research (pp. 1–9). Kennett Square, PA: American Association of Avian Pathologists.

[eva12515-bib-0079] Woolhouse, M. E. J. , Haydon, D. T. , & Antia, R. (2005). Emerging pathogens: The epidemiology and evolution of species jumps. Trends in Ecology & Evolution, 20, 238–244. 16701375 10.1016/j.tree.2005.02.009 PMC7119200

[eva12515-bib-0080] Woolhouse, M. E. J. , Taylor, L. H. , & Haydon, D. T. (2001). Population biology of multihost pathogens. Science, 292, 1109–1112. 11352066 10.1126/science.1059026

[eva12515-bib-0081] Woolhouse, M. E. J. , & Ward, M. J. (2013). Sources of antimicrobial resistance. Science, 341, 1460–1461. 24030495 10.1126/science.1243444

[eva12515-bib-0082] Xu, H. , Yao, Y. , Zhao, Y. , Smith, L. P. , Baigent, S. J. , & Nair, V. (2008). Analysis of the expression profiles of Marek's disease virus‐encoded microRNAs by real‐time quantitative PCR. Journal of Virological Methods, 149, 201–208. 18355930 10.1016/j.jviromet.2008.02.005

[eva12515-bib-0083] Yao, Y. , Zhao, Y. , Smith, L. P. , Watson, M. , & Nair, V. (2009). Novel microRNAs (miRNAs) encoded by herpesvirus of Turkeys: Evidence of miRNA evolution by duplication. Journal of Virology, 83, 6969–6973. 19403687 10.1128/JVI.00322-09 PMC2698521

[eva12515-bib-0084] Zhao, Y. , Xu, H. , Yao, Y. , Smith, L. P. , Kgosana, L. , Green, J. , … Nair, V. (2011). Critical role of the virus‐encoded microRNA‐155 ortholog in the induction of Marek's disease lymphomas. PLoS Pathogens, 7, e1001305. 21383974 10.1371/journal.ppat.1001305 PMC3044692

