# Supplementary material for: A phylogenomic analysis of Marek's disease virus reveals independent paths to virulence in Eurasia and North America
Source: Evol Appl. 2017 Sep 3;10(10):1091–101. doi: 10.1111/eva.12515 (PMC5680632; doi:10.1111/eva.12515)
Supplement: Supplementary file 1 [file EVA-10-1091-s001.docx]

**Supporting Information**

**Supplementary Table 1.** Sample information**.**

| **Sample** | **Year isolated** | **Accession code** | **Pathotype** | **Source details** | **Country** | **study** |
| --- | --- | --- | --- | --- | --- | --- |
| C12/130-10 | 1992 | FJ436096 | Hypervirulent | passage #2 | United Kingdom | Spatz et al. 2011 |
| C12/130-15 | 1992 | FJ436097 | Hypervirulent | (mildly attenuated) | United Kingdom | Spatz et al. 2011 |
| Md11 | 1977 | AY510475 | vv | passage #16 | US | Niikura et al. 2006; Witter et al. 1980; Witter & Fadly 1978 |
| Md5 | 1977 | AF243438 | vv | passage #3 | US | Tulman et al. 2000; Witter et al. 1980; Witter & Fadly 1978 |
| RB1B | 1981 | EF523390 | vv | passage #4 | US | Spatz et al. 2007a. Petherbridge et al. 2004, Schat et al. 1981,1982 |
| GX0101 | 2001 | JX844666 | vv | Field isolate | China | Su et al. 2012; Zhang & Cui 2005 |
| GA | 1964 | AF147806 | v | (highly attenuated) | US | Lee et al. 2000; Eidson & Schmittle 1968; Fukuchi et al. 1984; Gibbs et al. 1984; Purchase 1969 |
| CU-2 | 1968 | EU499381 | m | passage #14 | US | Spatz & Rue 2007; Smith & Calnek 1973 |
| CVI988 | 1969 | DQ530348 | m | passage #26 | Netherlands | Spatz et al. 2007b; Rispens et al. 1972 |
| 814 | 1986 | JF742597 | m | passage #20 | China | Zhang et al. 2012 |
| LMS | 2007 | JQ314003 | vv | passage #3 | China | Cheng et al. 2012 |
| 648A | 1994 | JQ806361 | vv+ | passage #10 | US | Spatz et al. 2012; Witter et al. 2005;  Witter 1997 |
| 584A | 1990 | EU627065 | vv+ | passage #80 (highly attenuated) | US | Spatz et al. 2008; Witter et al. 2005;  Witter 1997 |
| EU-1 | 1992 | MF431494 | Hypervirulent | <10 passages | Italy | Schumacher et al. 2002; Burgess, 2004.  Tischer et al. 2002 |
| Bd2 | 2015 | KU173119 | Hypervirulent | Field isolate (<5 passages) | US | Unpublished (This study) |
| Bf2 | 2015 | KU173118 | Hypervirulent | “ | US | “ |
| Bf1 | 2015 | KU173117 | Hypervirulent | “ | US | “ |
| Sd1 | 2015 | KU173116 | Hypervirulent | “ | US | “ |
| Sd2 | 2015 | KU173115 | Hypervirulent | “ | US | “ |
| MD70/13 | 1970 | MF431495 | v | “ | Hungary | “ |
| ATE2539 | 2000 | MF431493 | vv+ | “ | Hungary | “ |
| Polen5 | 2010 | MF431496 | Hypervirulent | “ | Poland | “ |

| Recombination event | Position in Md5 ref genome | ORFs | Affected sequences |
| --- | --- | --- | --- |
| 1 | 63203 – 116381 | MDV040-066 | Bf1, Bf2, Bd2, Sd1, Sd2 |
| 2 | 121564 - 128681 | MDV070-073 | GX0101, C12/130-10, -15 |

**Supplementary Table 2.** Details of recombinant regions and affected sequences.

**Supplementary Fig. 1.** Genetic variation across the alignment as depicted by Nei’s Pi of a 1Kb sliding window with 100bp interval length.

**
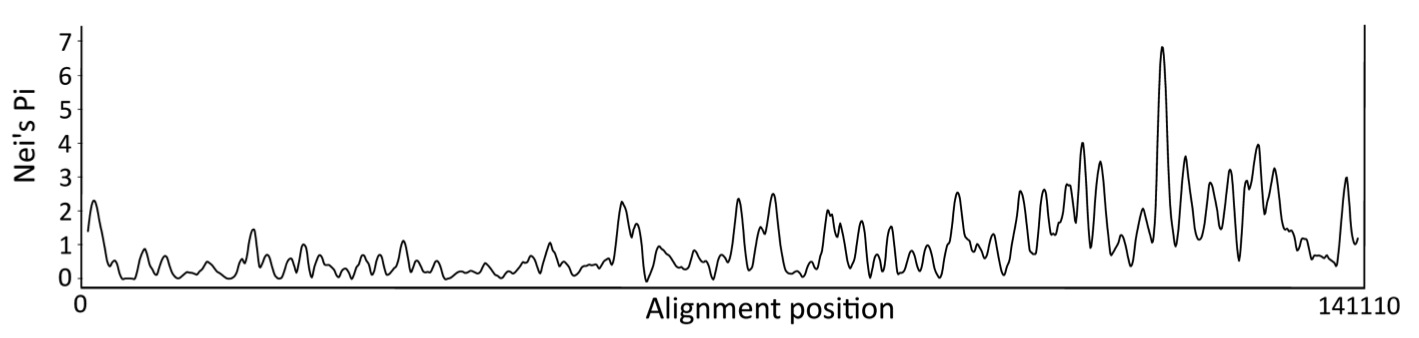
**

**Supplementary Fig. 2.** Impact of removal of highly attenuated genomes and recombination on topology and temporal signal in the data. Tree topologies from the ML analysis using a GTR + Γ_4_ substitution model and 1000 bootstrap replicates. Bootstrap support is indicated at each node (%). The midpoint-rooting for each tree is indicated with a black arrow. (a) All data; (b) GA and 584A removed; (c) recombinant regions, GA and 584A removed. Pathotype traits at tree tips are depicted according to the color scheme adopted in the main text.

**
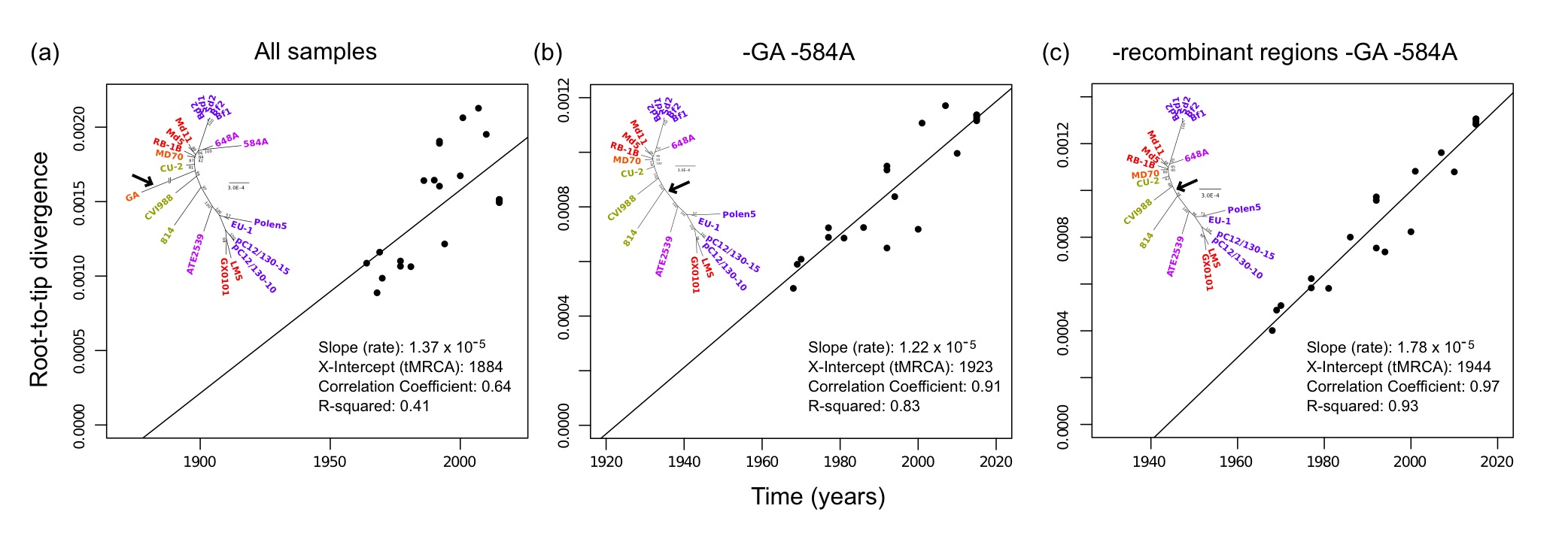
**

**Supplementary Fig. 3.** Amino acid substitutions for MDV068 (ICP27), MDV076 (Meq) and MDV084 (ICP4) mapped onto the backbone of the MDV phylogeny. Reversals are indicated in red, while putative parallel amino acid substitutions are inside a box. Code legend: [from amino acid][codon position in gene][to amino acid]

**Literature Cited**

Burgess, S.C. (2004). Marek’s disease lymphomas. In F. Davison & V. Nair (Eds.), Marek’s Disease: An Evolving Problem (pp. 98 -/111). London: Elsevier Academic Press.

Cheng, Y., Cong, F., Zhang, Y. et al. Genome sequence determination and analysis of a Chinese virulent strain, LMS, of Gallid herpesvirus type 2 (2012) Virus Genes 45: 56.

Eidson, C. S., & Schmittle, S. C. (1968). Studies on acute Marek's disease I. Characteristics of isolate GA in chickens. Avian diseases, 12(3), 467-476.

Fukuchi, K., Sudo, M., Lee, Y. S., Tanaka, A., & Nonoyama, M. (1984). Structure of Marek's disease virus DNA: detailed restriction enzyme map. Journal of virology, 51(1), 102-109.

Gibbs, C. P., Nazerian, K., Velicer, L. F., & Kung, H. J. (1984). Extensive homology exists between Marek disease herpesvirus and its vaccine virus, herpesvirus of turkeys. Proceedings of the National Academy of Sciences, 81(11), 3365-3369.

Lee, L. F., P. Wu, D. Sui, D. Ren, J. Kamil, H. J. Kung, and R. L. Witter. (2000). The complete unique long sequence and the overall genomic organization of the GA strain of Marek’s disease virus. Proc. Natl. Acad. Sci. U.S.A. 97:6091–6096.

Niikura, M., Dodgson, J., & Cheng, H. (2006). Direct evidence of host genome acquisition by the alphaherpesvirus Marek’s disease virus. Archives of virology, 151(3), 537-549.

Petherbridge, L., Brown, A. C., Baigent, S. J., Howes, K., Sacco, M. A., Osterrieder, N., & Nair, V. K. (2004). Oncogenicity of virulent Marek's disease virus cloned as bacterial artificial chromosomes.Journal of virology, 78(23), 13376-13380.

Purchase, H. G. (1969). Immunofluorescence in the Study of Marek's Disease I. Detection of Antigen in Cell Culture and an Antigenic Comparison of Eight Isolates. Journal of virology,3(6), 557-565.

Rispens, B. H., van Vloten, H., Mastenbroek, N., Maas, H. J. & Schat, K. A. (1972). Control of Marek's disease in the Netherlands. I. Isolation of an avirulent Marek's disease virus (strain CVI 988) and its use in laboratory vaccination trials. Avian Dis 16, 108–125.

Schat, K. A., Calnek, B. W., & Fabricant, J. (1982). Characterisation of two highly oncogenic strains of Marek's disease virus 1 2. Avian Pathology, 11(4), 593-605.

Schat, K. A., Calnek, B. W., Fabricant, J., & Abplanalp, H. (1981). Influence of oncogenicity of Marek’s disease virus on evaluation of genetic resistance. Poultry science, 60(12), 2559-2566.

Schumacher, D., Tischer, B.K., Teifke, J.P., Wink, K. & Osterrieder, N. (2002). Generation of a permanent cell line that supports efficient growth of Marek’s disease virus (MDV) by constitutive expression of MDV glycoprotein E. Journal of General Virology, 83, 1987 - 1992.

Smith, M. W. & B.W. Calnek. (1973). Effect of virus pathogenicity on antibody production in Marek’s disease. Avian Dis. 17, 727–736.

Spatz, S. J., Zhao, Y., Petherbridge, L., Smith, L. P., Baigent, S. J., & Nair, V. (2007a). Comparative sequence analysis of a highly oncogenic but horizontal spread-defective clone of Marek’s disease virus. Virus Genes, 35(3), 753-766.

Spatz, S. J., Petherbridge, L., Zhao, Y., & Nair, V. (2007b). Comparative full-length sequence analysis of oncogenic and vaccine (Rispens) strains of Marek's disease virus. Journal of General Virology,88(4), 1080-1096.

Spatz, S. J., & Rue, C. A. (2008). Sequence determination of a mildly virulent strain (CU-2) of Gallid herpesvirus type 2 using 454 pyrosequencing. Virus Genes, 36(3), 479-489.

Spatz, S.J., Rue, C., Schumacher, D. et al. (2008) Clustering of mutations within the inverted repeat regions of a serially passaged attenuated gallid herpesvirus type 2 strain. Virus Genes 37: 69.

Spatz SJ, Smith LP, Baigent SJ, Petherbridge L, Nair V (2011) Genotypic characterization of two bacterial artificial chromosome clones derived from a single DNA source of the very virulent gallid herpesvirus-2 strain C12/130. J Gen Virol 92:1500–1507

Spatz, S.J., Volkening, J.D., Gimeno, I.M. et al. (2012) Dynamic equilibrium of Marek’s disease genomes during in vitro serial passage. Virus Genes 45: 526.

Su, S., Cui, N., Cui, Z., Zhao, P., Li, Y., Ding, J., & Dong, X. (2012). Complete Genome Sequence of a Recombinant Marek’s Disease Virus Field Strain with One Reticuloendotheliosis Virus Long Terminal Repeat Insert. Journal of Virology, 86(24), 13818–13819.

Tischer, B. K., Schumacher, D., Beer, M., Beyer, J., Teifke, J. P., Osterrieder, K., ... & Osterrieder, N. (2002). A DNA vaccine containing an infectious Marek’s disease virus genome can confer protection against tumorigenic Marek’s disease in chickens. Journal of general virology, 83(10), 2367-2376.

Tulman, E. R., Afonso, C. L., Lu, Z., Zsak, L., Rock, D. L., & Kutish, G. F. (2000). The genome of a very virulent Marek's disease virus. Journal of Virology, 74(17), 7980-7988.

Witter, R. L. (1997). Increased virulence of Marek’s disease virus field isolates. Avian Diseases, 41:149–163

Witter, R. L., B. W. Calnek , C. Buscaglia , I. M. Gimeno & K. A. Schat (2005). Classification of Marek's disease viruses according to pathotype: philosophy and methodology, Avian Pathology, 34:2, 75-90.

Witter, R. L., Sharma, J. M., & Fadly, A. M. (1980). Pathogenicity of variant Marek's disease virus isolants in vaccinated and unvaccinated chickens. Avian Diseases, 210-232.

Witter, R. L., & A. M. Fadly. Marek's disease virus isolates with increased pathogenicity for immunized chickens. Proc. European Econ. Community Symp. on Resistance and Immunity to Marek's disease, Berlin, West Germany. 1978 (In press).

Zhang, F., Liu, C. J., Zhang, Y. P., Li, Z. J., Liu, A. L., Yan, F. H., ... & Cheng, Y. (2012). Comparative full-length sequence analysis of Marek’s disease virus vaccine strain 814. Archives of virology,157(1), 177-183.

Zhang Z, Cui Z. Isolation of recombinant field strains of Marek’s disease virus integration with reticuloendotheliosis virus genome fragments. Sci China C-Life Sci, 2005, 48: 81―88
